# Rare Metastatic Sites of a Lung Adenocarcinoma

**DOI:** 10.7759/cureus.2819

**Published:** 2018-06-17

**Authors:** Rui Ya Soh, Chia Ming Ho, Kai Ling Soo, Su Ying Low

**Affiliations:** 1 General Medicine, Sengkang General Hospital, Singapore, SGP; 2 Diagnostic Radiology, Singapore General Hospital, Singapore, SGP; 3 Anatomical Pathology, Singapore General Hospital, Singapore, SGP; 4 Respiratory and Critical Care Medicine, Singapore General Hospital, Singapore, SGP

**Keywords:** metastasis, lung adenocarcinoma, thyroid transcription factor-1

## Abstract

We report a rare case of a lung adenocarcinoma presenting with chylothorax and metastases to the common bile duct and the rectum. From the radiological and endoscopic appearance of the tumors, the main differential diagnoses were metastatic lung cancer to multiple rare extra-thoracic sites and multiple synchronous primary oncological malignancies. Pathological examination of the biopsies with positive immunohistochemical staining for thyroid transcription factor-1 (TTF-1) played an important role in confirming metastatic pulmonary adenocarcinoma.

## Introduction

Lymph nodes, liver, adrenal glands, brain, bone and other parts of the lung and pleura are common metastatic sites of lung cancer. Although pleural effusion is a frequent presentation in lung cancer, chylothorax is seldom seen in primary lung malignancy in the absence of cardiothoracic surgery [1]. Malignancy-related biliary obstruction is usually seen in primary biliary cancer or cancer involving head of pancreas. Metastasis from a non-biliary and non-gastrointestinal tract cancer to the common bile duct is rare and is an unusual presentation for lung cancer [2]. Likewise, the colorectal mucosa is an uncommon site for metastasis and when present, is usually related to peritoneal seeding or direct invasion from an intra-abdominal malignancy [3-5]. We hereby describe a unique case of a metastatic lung cancer with simultaneous involvement of the common bile duct, rectum and chylothorax at initial presentation.

## Case presentation

**Figure 1:**
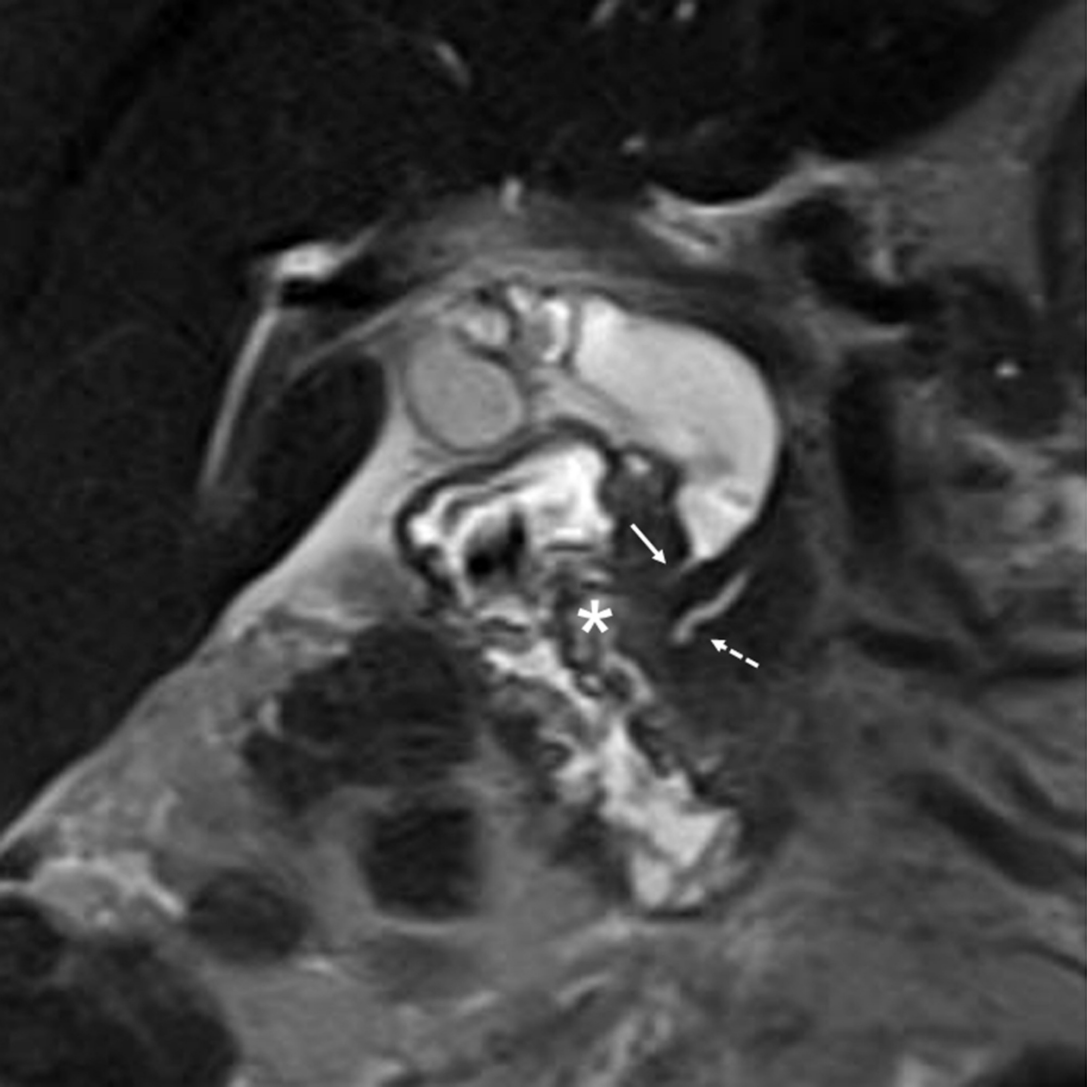
Magnetic resonance cholangiopancreatography (MRCP). The cystic duct and common bile duct are markedly distended with a smooth and short narrowing (solid arrow) at the distal aspect. No biliary stone is identified. The main pancreatic duct (dotted arrow) is not involved. Along the distal common duct, there is an area of ill-defined increased T2-weighted signal. The adjacent duodenal wall (*) shows prominently increased T2-weighted signal that reflects oedema which can be reactive to an infiltrative disease.

**Figure 2:**
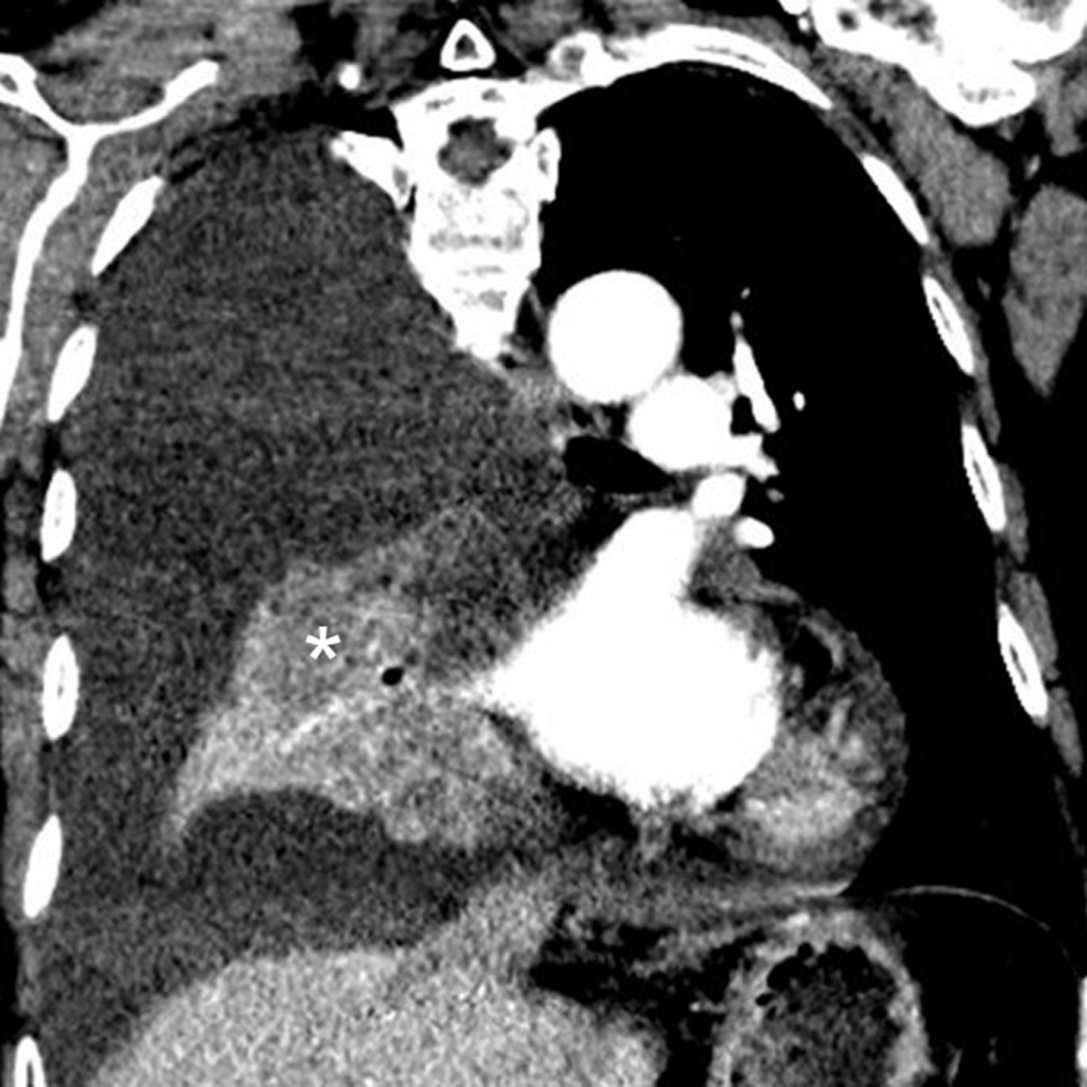
Computed tomography (CT) of the chest confirms a large right pleural effusion with collapse of the middle and lower lobe. The bulging nodular hypoenhancing mass (*) in the right lower lobe is suspicious for primary bronchogenic malignancy.

A 78-year-old non-smoker Chinese female presented with a six-month duration of progressive breathlessness. This was associated with cough, poor appetite and weight loss over the last one month. She had reduced air entry on the right chest with stony dullness on percussion. Chest radiograph showed a right moderate effusion. Thoracocentesis drained milky fluid which was biochemically in keeping with chylothorax (pH 7.7; triglycerides, 3.18 mmol/l; total cholesterol, 2.44 mmol/l; lactate dehydrogenase, 632 U/L; protein, 43 g/l and glucose, 6.2 mmol/l). The fluid had no malignant cells or pathogens. Blood biochemistry showed obstructive liver function (alkaline liver phosphatase, 899 U/L; alanine aminotransferase, 51 U/L; aspartate aminotransferase, 79 U/L and total bilirubin, 14 umol/l). Computed tomography (CT) scan revealed a right lower lobe lung mass with a right pleural effusion (Figure 1), rectum thickening, prominent common bile duct and a moderate left hydronephrosis with soft tissue non-opacification in the left mid-ureter. Magnetic resonance cholangiopancreatography (MRCP) (Figure 2) showed suspicion of a common bile duct stricture with biliary tree dilatation and an abrupt change in calibre at the distal common bile duct without any stone or soft tissue mass seen in the biliary and pancreatic region. In view of the radiological and clinical findings, main differential diagnosis was metastatic lung cancer to multiple extra-thoracic sites versus multiple synchronous primary oncological malignancies.

**Figure 3:**
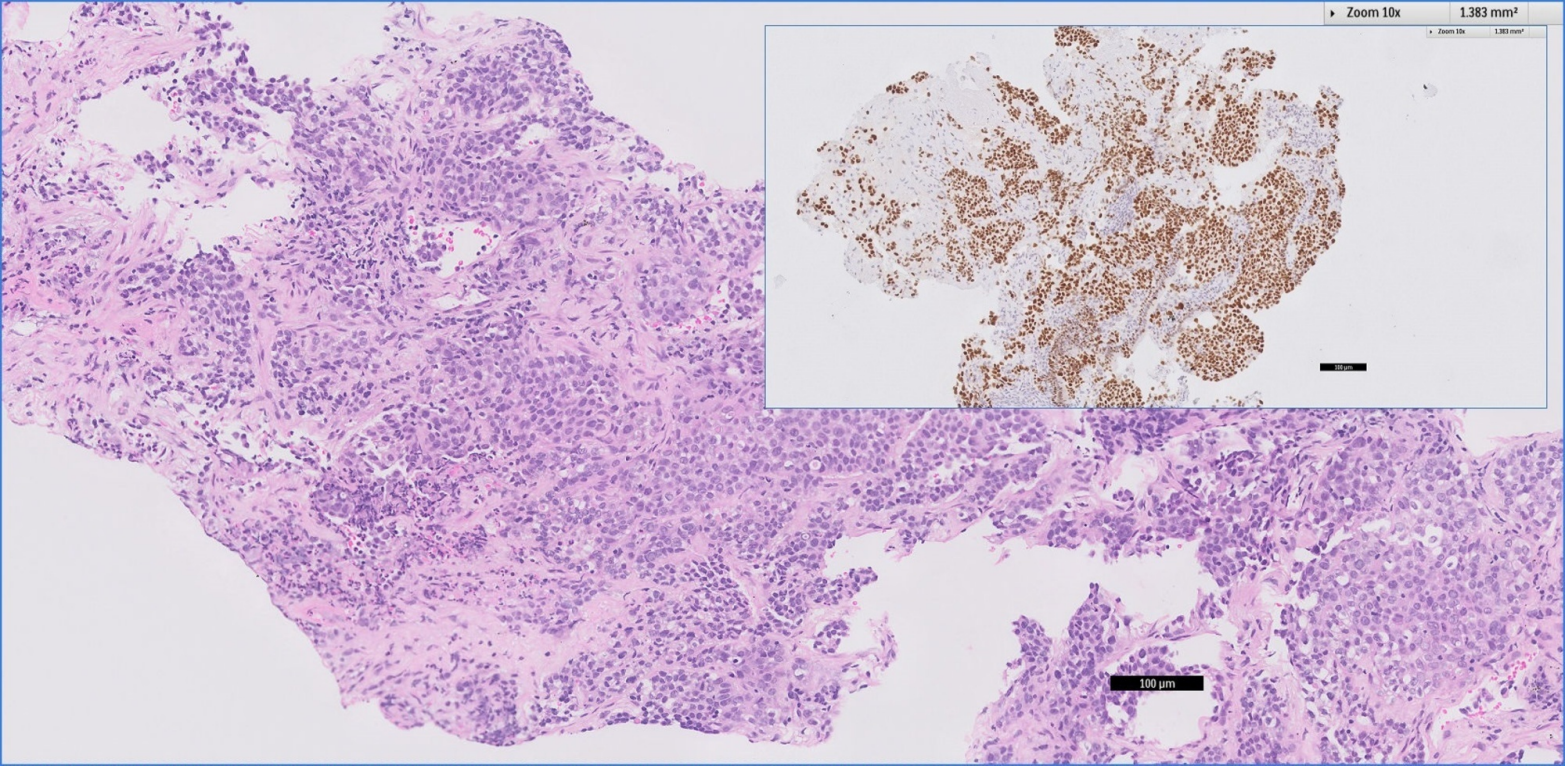
Transthoracic needle biopsy of the right lung lesion. This shows a non-small cell carcinoma (Hematoxylin and Eosin stain, x100) with diffuse strong nuclear TTF-1 expression (inset figure) in keeping with an adenocarcinoma of pulmonary origin.

**Figure 4:**
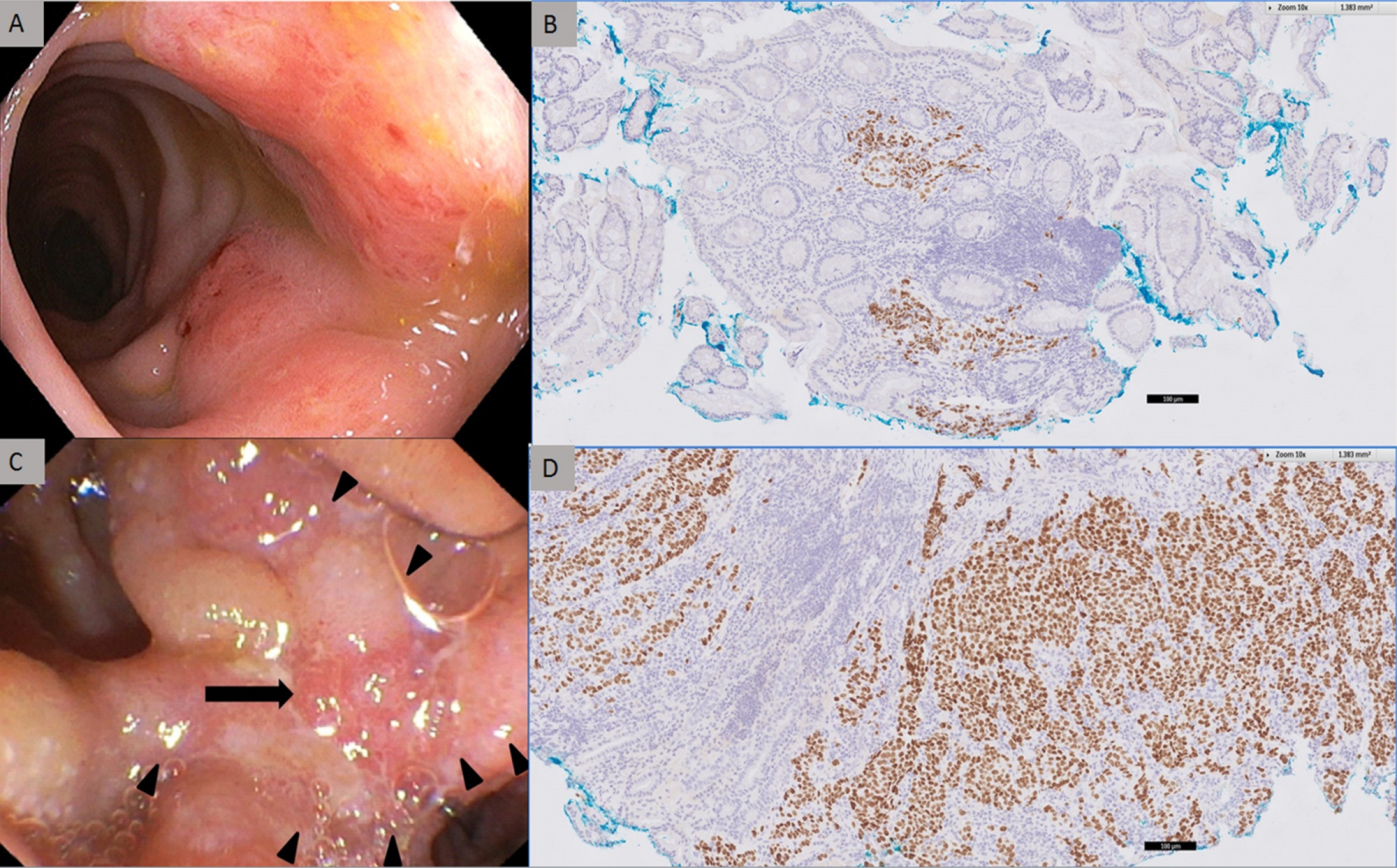
Endoscopy images of the metastatic tumors and their corresponding histology showing positive immunohistochemical staining with TTF-1. (A) Colonoscopy shows a circumferential lateral-spreading tumor located 15 cm from the anal verge. (B) Endoscopic recto-sigmoid biopsy shows a poorly differentiated carcinoma of similar immunohistomorphology to that of the lung lesion (TTF-1 immunohistochemical stain, x100). (C) Endoscopic retrograde cholangiopancreatography. The arrowheads indicate the periampullary tumor extending to the duodenal margins. The arrow indicates the papilla of the common bile duct. (D) Endoscopic duodenal biopsy shows a poorly differentiated carcinoma of similar immunohistomorphology to that of the lung lesion (TTF-1 immunohistochemical stain, x100).

CT-guided core needle biopsy of the lung mass revealed pulmonary adenocarcinoma with diffuse strong nuclear immunohistochemical staining for thyroid transcription factor-1 (TTF-1) (Figure 3). Epidermal growth factor receptor mutational analysis was negative. Colonoscopy showed a circumferential rectal tumor with biopsies (Figure 4A-4B) proving adenocarcinoma in keeping with lung metastasis (positive immunohistochemical staining for TTF-1; negative for caudal-related homeobox 2 (CDX2)). Endoscopic retrograde cholangiopancreatography showed a short distal common bile duct obstruction by a periampullary tumor extending to the duodenal margins (Figure 4C). Biliary stent was inserted with successful good drainage. Cytobrushing and biopsies (Figure 4D) revealed poorly-differentiated adenocarcinoma of lung origin (positive immunohistochemical staining for TTF-1, Napsin-A, cytokeratin (CK)7, CK19, mucicarmine 1; negative for CK20, chromogranin, synaptophysin and carbonic anhydrase 19-9). Invasive biopsy was not performed for the ureter lesion but impression at a multi-disciplinary tumor board discussion was that it was clinical-radiologically in keeping with a synchronous metastatic lung lesion.

## Discussion

Very few extra-pulmonary lung cancer metastases to the gastrointestinal [3-5], biliary [2] or genitourinary tract [6] have been reported. Our case of a lung adenocarcinoma with chylothorax and biopsy-proven synchronous rectal and common bile duct metastases is a rare presentation. As illustrated in this case (Figure 4A, 4C), the endoscopic morphology of the metastatic tumors cannot be differentiated from primary malignancies. The coordinated expression of specific positive and negative immunohistochemical stains formulates characteristic immunophenotype which is used to identify the origin of the carcinoma. CK7 and TTF-1 are highly specific for lung carcinomas while CK20 and CDX2 are commonly expressed in intestinal carcinomas [7]. This rare presentation of biopsy-proven synchronous rectal and common bile duct metastases highlights the important role of immunohistochemical staining in identifying lung adenocarcinoma presenting as metastases in uncommon sites.

Chylothorax occurs when there is a disruption of the thoracic duct or its tributaries, resulting in leakage of chyle into the pleural space. In the absence of traumatic thoracic ductal injury from lung resection surgery, occurrence of chylothorax in lung cancer is not common. In view of the size and location of the tumor in our patient, the possible cause may be direct invasion or extrinsic compression of the thoracic duct by the tumor [1]. Other causes of lung malignancy-related chylothorax include late complication of thoracic radiotherapy and superior vena cava syndrome where obstruction in the superior vena cava causes the venous pressure to exceed the thoracic ductal pressure, resulting in chyle leakage from the pleural lymphatic ducts [1]. These are less likely causes in our patient who did not have previous radiotherapy and physical signs of superior vena cava syndrome.

Distant metastasis of lung cancer represents a late stage disease with poor prognosis reported in a case series of colonic metastasis [7]. Our patient demised within a month of diagnosis of pneumonia. Due to the rarity, there is no definite literature explaining how lung cancer selectively metastasizes to certain remote organs. Several hypotheses have been proposed. The cancer stem cell theory describes a subset of highly metastatic tumor cell variant [8-9] while the “seed and soil theory” emphasizes on tumor-stromal affinity between tumor cells and the specific microenvironment of the metastatic niche-organ [5, 9-10]. The lymphatic and hematogenous dissemination theory describes how metastasis follows the vascular and lymphatic drainage from the primary tumor to affect the first organ encountered [10]. The angiogenic switch theory suggests various triggering factors that promote tumor angiogenesis and neovascularization [5, 8] while the epithelial-to-mesenchymal transition theory describes how tumor cells are transformed to highly mobile cells to promote metastasis [5, 9]. Better understanding of the metastatic process may reveal more therapeutic targets for future oncological treatment.

## Conclusions

Immunohistochemical staining plays an important role in identifying metastasis in uncommon sites. This has a strong impact on accurate staging of malignancy which will in turn, implicate prognostication and management.
